# Polypyrrole-Stabilized Polypeptide for Eco-Friendly Supercapacitors

**DOI:** 10.3390/ijms24032497

**Published:** 2023-01-27

**Authors:** Zhe Li, Kuan Hu, Zhou Li, Cong Li, Yulin Deng

**Affiliations:** 1 School of Medical Technology, Institute of Engineering Medicine, Beijing Institute of Technology, Beijing 100081, China; 2 Institute of Materia Medica, Chinese Academy of Medical Sciences & Peking Union Medical College, Beijing 100050, China; 3 Beijing Institute of Nanoenergy and Nanosystems, Chinese Academy of Sciences, Beijing 101400, China; 4 School of Life, Beijing Institute of Technology, Beijing 100081, China

**Keywords:** eco-friendly, degradable, peptide, supercapacitor, pyrrole

## Abstract

As an energy storage technology, supercapacitors (SCs) have become an important part of many electronic systems because of their high-power density, long cycle life, and maintenance-free characteristics. However, the widespread development and use of electronics, including SCs, have led to the generation of a large amount of e-waste. In addition, achieving compatibility between stability and biodegradability has been a prominent challenge for implantable electronics. Therefore, environmentally friendly SCs based on polypyrrole (PPy)-stabilized polypeptide (FF) are demonstrated in this study. The fully degradable SC has a layer-by-layer structure, including polylactic acid/chitosan (PLA–C) support layers, current collectors (Mg), FF/PPy composite layers, and a polyvinyl alcohol/phosphate buffer solution (PVA/PBS) hydrogel. It has the advantages of being light, thin, flexible, and biocompatible. After 5000 cycles in air, the capacitance retention remains at up to 94.7%. The device could stably operate for 7 days in a liquid environment and completely degrade in vitro within 90 days without any adverse effect on the environment. This work has important implications for eco-friendly electronics and will have a significant impact on the implantable biomedical electronics.

## 1. Introduction

A supercapacitor (SC) is a type of energy storage device and has a similar electrochemical performance to traditional capacitors or batteries [1,2,3,4]. It has the characteristics of high-power density [5,6], long cycle life [7], being maintenance-free [8,9], and so on [10]. However, the upgrading of electronics including SCs produces a large amount of electronic waste (e-waste) [11,12]. According to the United Nations, e-waste from outdated electronics is projected to grow to 74.7 million tons by 2030. E-waste not only consume lots of space in landfill sites due to their extremely long degradation periods [13], but also cause serious environmental pollution in the degradation process [14]. Therefore, it is urgent to develop transient electronics to solve the above-mentioned problems of SCs.

In recent years, scientists extensively studied a variety of degradable materials and developed types of transient SCs [15,16]. After performing specific functions, these devices can be completely dissolved in vivo or in the environment and produce bio-harmless by-products. They have broadened potential applications in green electronics, implantable devices, personalized medicines, military security, and other fields [17,18,19]. For sensors, the performance of stable energy storage is critical. Good stability usually means that the device is composed of materials with stable chemical groups, while degradability (including controlled degradation) often requires weak bonds. It is a big challenge for degradable SCs to find the optimal critical point between controllable degradation and anti-aging properties.

Diphenylalanine (FF) is a type of aromatic dipeptide derived from the self-assembly of beta-amyloid protein [20,21,22]. It has excellent mechanical properties, electrical properties, and biosafety [23,24,25]. It is employed in biomedicine, nanoscience, electronics, and other fields [26,27,28]. In previous works, the FF-based SCs achieved efficient and stable energy storage, providing a viable solution for portable, distributed implantable, or wearable electronics [21,29]. However, the capacities and the stabilities need to be improved, and the flexibility, biocompatibility, and degradability of the SCs need to be further optimized.

In this work, based on FF and pyrrole (Py), FF films stabilized by electrochemical-deposited polypyrrole (FF/PPy) and FF–Py copolymer films were designed and prepared. The performance of films was studied and compared using three-electrode systems and assembled all-solid eco-friendly SCs. The optimal FF/PPy-based SC included two polylactic acid–chitosan (PLA–C) support layers, a couple of current collectors (Mg), two pieces of active materials (FF/PPy films), and a polyvinyl alcohol/phosphate buffer solution (PVA/PBS) hydrogel. It not only shows good stability, but also has excellent biocompatibility and degradability. After 5000 cycles in air, the capacitance retention remains at up to 94.7%. The SC can work stably for 7 days in a liquid environment and disappear completely within 90 days. It will promote the development of transient electronics and shows great potentials in both the environmental and biomedical fields.

## 2. Results

### 2.1. Fabrication and Characterization of FF/PPy and FF–Py Copolymer Films 

**Figure 1:**
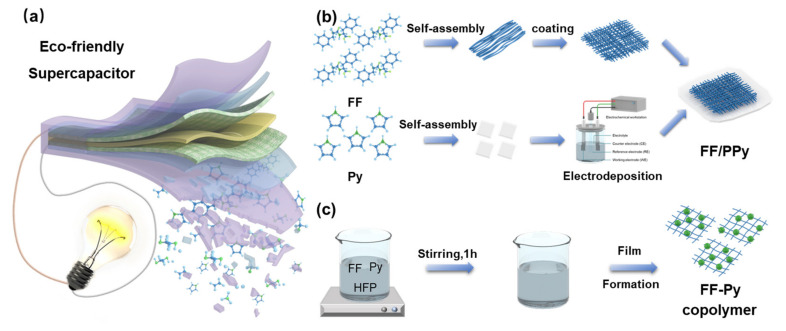
Schematic illustration of degradable supercapacitor (**a**) and the fabrication of FF/PPy (**b**) and FF–Py copolymer (**c**) films.

In this study, an eco-friendly supercapacitor (SC) was fabricated based on FF and Py. The SC can light a LED and will disappear completely within 90 days (Figure 1a and Figure S4). At first, two kinds of active materials for SCs were prepared. Figure 1b shows the fabrication of FF/PPy. FF nanotubes were self-assembled into a cross-linked network on Mg/PLA–C plate at first, which provided a large area for the further deposition of PPy. Then, the Py monomer was polymerized into PPy and electrochemically deposited on the FF network. Finally, the active material FF/PPy film was prepared. Figure 1c shows the preparation of the FF–Py copolymer. It was supplied as the other type of active material. FF nanotubes were uniformly dispersed in 75% alcohol with hexafluoroisopropanol (HFIP) and mixed with Py monomer. After stirring for one hour, the blend was smeared on the Mg/PLA–C plate, and it was named as the FF–Py copolymer. These two fabricated films (FF/PPy and FF–Py copolymer) were selected for further study in the following experiments.

**Figure 2:**
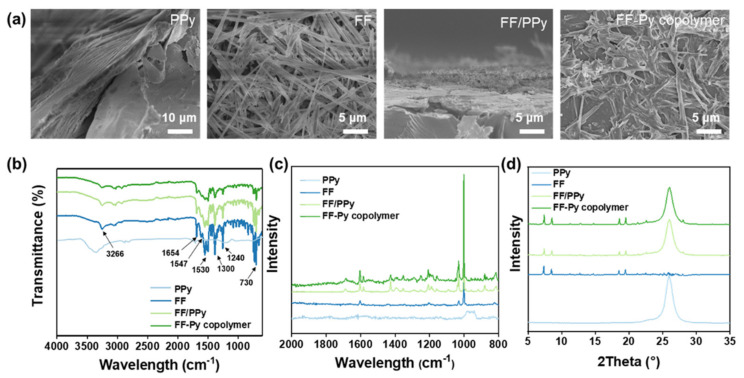
Characterization and electrochemical property of PPy, FF, FF/PPy, and FF–Py copolymer. (**a**) SEM images; (**b**) FTIR spectrum; (**c**) Raman spectrum; (**d**) XRD pattern.

Various FF/PPy films were prepared by changing the deposition times of PPy. The micromorphologies of PPy, FF, and FF/PPy were characterized by scanning electron microscope (SEM). The pure PPy has layers of stacked sheets (Figure 2a). As shown in Figure 2b, the self-assembled FF haves a nanotube-like structure and are interwoven with each other as a network. After electrodepositing PPy for different cycles, the morphology of FF varies greatly (Figure S1a–e). After 50 cycles of deposition, the PPy covers the FF nanotubes, resulting in a densely cross-linked reticular structure, which consists of intersecting nanofibers, pores, and microfibers. The large size fibers are formed by the self-organization of the nanofibers, which commonly appears in the assemblies of peptide as revealed by previous reports [30,31,32]. It was anticipated that the enormous porous structure would provide enough surface area for electrolyte infiltration. As the deposition cycles increase, more and more PPy nanosheets are formed in the skeleton of the FF network. However, as the deposition cycles reaches 200 cycles, most of the FF nanotubes are packaged and a wide range of PPy nanosheets are attached to the surface.

In order to verify FF had been self-assembled into the nanotube structure stably, the assembled nanotubes were dropped onto pretreated silicon wafers, sprayed with gold, and then observed by scanning electron microscopy (SEM). As shown in Figure 2a, the structure of the nanotube is clear and regular, with dense fibers interlaced in a network. The diameter of a single FF nanotube is about 200–500 nm, and the length can reach more than 50 μm. Similarly, the morphology of FF/PPy and the FF–Py copolymer were observed. FF/PPy has a multilayer lamellar structure, which proves that FF and PPy are layered the on Mg/PLA–C substrate. However, the FF–Py copolymer has a massive structure, whose uniformity is inferior to FF/PPy.

The active materials were further characterized by Fourier transform infrared spectroscopy (FTIR), Raman, and X-ray diffraction (XRD). The potassium bromide solid tablet method was used in FTIR, and the scanning range was 4000–600 cm^−1^. The Raman scanning range was 800~2000 cm^−1^. The XRD scanning speed was 2°·min^−1^, scanning range was 5°~35°, and the λ was 1.5418 Å under a CuKα ray.

FTIR and Raman spectroscopy were applied to further characterize various active materials. FTIR provided information on component chemistry and secondary structure. The spectrum of the composition (Figure 2b, Figures S1g and S2f) contains strong bands of amide I at 1654 cm^−1^ and amide II at 1547 cm^−1^ and 1530 cm^−1^, indicating that FF is an α-helical structure. In the range of 3700–3300 cm^−1^, there are amine bi-peaks in moderate intensity and the stretching vibration peak of O-H. The N-H stretching frequencies at 3319 cm^−1^ and 3266 cm^−1^ correspond to the hydrogen bond network. The peak at 1600–1450 cm^−1^ is the skeleton vibration peak of C=C on benzene. In addition, the stretching vibration peak of the C=O bond also exists around 1600 cm^−1^ as well, while at ~1300 cm^−1^, there is the stretching vibration peak of C-O on peptide. Furthermore, the peak near 730 cm^−1^ is attributed to the out-of-plane bending vibration of C-H on the benzene ring of FF. As shown in Figure S1g, as the eletrochemical deposition cycles of PPy increases to 150, the content of PPy on the FF/PPy film increases, the stretching vibration peak of C-N becomes stronger around 1240 cm^−1^, and the out-of-plane bending vibration of C-H on the benzene ring has a weaker strength. In Figure S2f, when the ratio of FF to Py is 1:1, there is the strongest out-of-plane bending vibration peak of C-H in the range of 1250–1000 cm^−1^. Compared with the Raman spectra of FF, FF/PPy and the FF–Py copolymer have similar absorption peaks in location, shape, and relative intensity. The results show that FF does not react with PPy or Py to form new functional groups during the preparation of FF/PPy and FF–Py copolymer films. Raman spectra show a peak at 1652 cm^−1^, indicating a helical structure of the individual peptide, which is consistent with FTIR spectra (Figure 2c). Figure 2d shows the XRD patterns of various active materials. With the peaks at 2θ of 7.59° and 19.75°, it indicates that the introduction of Py or PPy does not destroy the structure of FF, which further proves that FF only physically reacts with Py or PPy.

The electrochemical behaviors of FF/PPy films were investigated with cyclic voltammetry in 1.0 M aqueous KCl electrolyte. Platinum (Pt) electrode was applied as the counter electrode and the saturated calomel electrode was employed as reference electrode in the three-electrode system. The CV curves prove the great capacitive performance of the prepared active materials (Figure S1h). The area of CV curves increases with the electrodeposition up to 150 cycles and it shows the highest capacitance of 42 mF·cm^−2^. Then the area of CV curves decreases slightly at 200 cycles. It indicates that 150 cycles is a preferred parameter to prepare FF/PPy (Figure S1i). As a result, FF/PPy film with the electrodeposition of 150 cycles was selected for further study.

As shown in Figure 1b, the FF–Py copolymer was prepared with three different ratios and coated on the Mg/PLA–C plate. When FF is blended with Py in HFIP and the solvent evaporated, the copolymer assembles into nanosheets (Figure S2b). When the Py ratio increases to 1:2, the FF–Py copolymer turns into a blocky structure (Figure S2c). When FF is in the majority (2:1), the blend of FF–Py assembles into a jumble of massive structures (Figure S2d). 

The electrochemical behaviors of the FF–Py copolymer were also investigated with CV scanning in 1.0 M aqueous KCl electrolyte. Pt electrode was applied as the counter electrode and saturated calomel electrode was employed as reference electrode in the three-electrode system. Figure S2g shows the CV curves of FF–Py with three different ratios at 100 mV·s^−1^; the data represent different capacitive behaviors of the as-prepared samples. As shown in the results, the FF–Py copolymer at 1:1 has the largest specific capacitance (35 mF·cm^−2^) in three ratios (Figure S2h). As a consequence, the FF–Py copolymer film with the ratio of 1:1 was selected for the following experiment. 

### 2.2. Comparisons of Two Types of Active Materials

**Figure 3:**
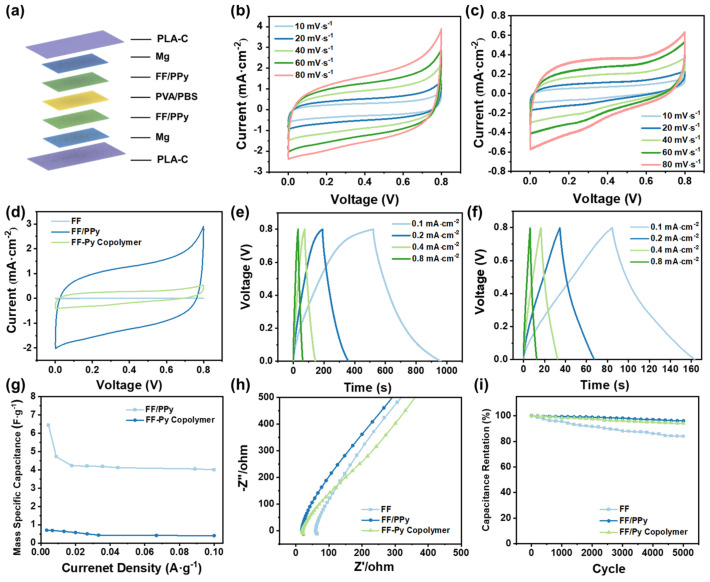
Electrochemical performance of all-solid SCs based on FF films, FF/PPy film with 150 cycles of PPy deposition, and FF–Py copolymer films with a ratio of 1:1. (**a**) Structure schematic of the FF/PPy-based SCs; (**b**–**d**) CV curves of FF/PPy-based SCs (**b**) and the SCs using FF–Py copolymer film (**c**) at different scan rates, respectively; (**d**) comparison of SCs based on FF, FF/PPy, and FF–Py copolymer films; (**e**) GCD curves of FF/PPy-based SCs at different current densities; (**f**) GCD curves of the SCs using FF–Py copolymer film at different current densities; (**g**) mass specific capacitances variation of SCs based on FF/PPy and FF–Py copolymer films with different scan rates; (**h**) Nyquist plot reflecting the impedance characteristics of SCs; (**i**) cycle stability test with the current density of 0.2 mA·cm^−2^ at 0.8 V.

Based on above results, we further compared the electrochemical performance of the all-solid SCs based on FF/PPy films with 150 cycles electrodeposition and the FF–Py copolymer with the ration of 1:1. The all-solid FF/PPy and FF–Py copolymer-based SCs were fabricated using PVA/PBS hydrogel as a solid electrolyte. Taking the FF/PPy-based SC for an example, the structure schematic is shown in Figure 3a. In Figure 3b,c, the CV curves of SCs based on FF/PPy and the FF–Py copolymer at scan rates from 10 mV·s^−1^ to 80 mV·s^−1^ are shown studied, respectively. It can be seen that the peak current increases with the addition of scanning speed. As shown in Figure 3d, the CV area of FF/PPy is obviously larger than that of pure FF and the FF–Py copolymer at 50 mV·s^−1^. Meanwhile, the discharging time of FF/PPy-based SCs (Figure 3e) are obviously longer than that of SCs based on FF–Py copolymer films (Figure 3f) at the same current density. Both the GCD and CV results prove that FF/PPy films have a better electrochemical performance than FF–Py copolymer films. According to Equations (1) and (2), the specific capacitances versus different current densities are plotted in Figure 3g and Figure S3a. These results indicate that the capacitance of the devices decreases with the decrease in the potential window. As shown in Figure 3g, the mass specific capacitances of FF/PPy-based SCs are higher than those using the FF–Py copolymer, which can achieve 4 F·g^−1^ at the current density of 0.1 A·g^−1^. In addition, the result of areal specific capacitances of both types of SCs further verify that the FF/PPy-based SCs have a better capacity of energy storage. 

For further validation of the great capacitance performance of FF/PPy-based SCs, the Nyquist plot reflecting the impedance characteristics is recorded in Figure 3h. All spectra display a sloping line in the high frequency range. The equivalent series resistances (Rs) are about 13.3, 20.0, and 60.0 Ω for FF/PPy, the FF–Py copolymer, and FF, respectively. The differences between SCs based on FF/PPy and FF–Py copolymer films may be caused by the following reasons. Firstly, the blend of FF and Py could not provide enough electroactivity while the PPy is sufficient to cover all FF film, resulting in a continuous and strong adhesion on the Mg/PLA plate. Secondly, the excellent contact of FF and PPy leads to sufficient electron transfer. In short, FF could provide a continuous and intertwined adhesion layer for PPy, which enhances both conductivity and capacitance of the FF/PPy-based SC. A cycle stability test was carried out to evaluate the stability of FF, FF/PPy, and the FF–Py copolymer. As depicted in Figure S3b,c, the cycle stability of the SCs are tested with GCD cycling at a current density of 0.8 mA·cm^−2^. The capacitance retention after 5000 cycles for FF/PPy, the FF–Py copolymer, and FF are about 94.8%, 93.1%, and 84.2%, respectively (Figure 3i). These results indicate that FF/PPy shows the best cycle stability, and it was chosen for further study. Furthermore, the FF/PPy-based SCs exhibit good electrochemical performance and excellent biodegradability as compared to the recently developed peptide and hybrid electrode-based SCs. The electrochemical activity of peptide-based nanomaterials and hybrids electrodes are compared with FF/PPy film in Table S1 [11,16,21,29,33,34,35,36,37,38,39,40,41,42,43,44].

### 2.3. Capacitive Performance and Biodegradability of SC in the Liquid Environment In Vitro

**Figure 4:**
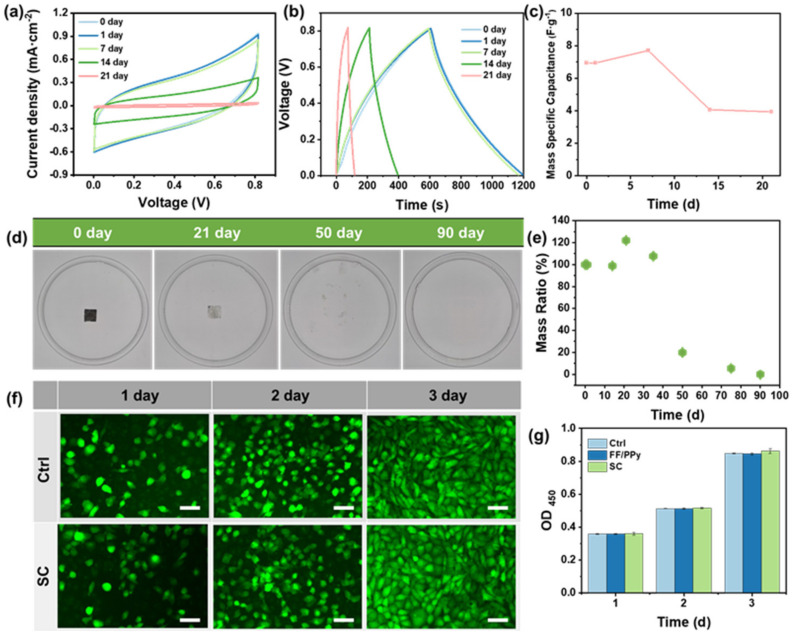
Capacitive performance and biodegradability of SCs in the liquid environment in vitro. Electrochemical performance test of SCs for a short-term in PBS at 37 °C (**a**–**c**). The current density in (**b**) is 0. 2 mA·cm^−2^; (**c**) the variation of mass specific capacitance during device’s degradation; (**d**) in vitro degradation of SCs in 1× PBS buffer in a cell culture dish (inner diameter: 35 mm) at room temperature; (**e**) the mass variation of SCs during degradation; (**f**) biocompatibility of SCs. Attachment, proliferation, and morphology of the L929 cells at different times. Scale bars: 100 µm; (**g**) the viabilities of L929 cells after being cultured for 3 days. All data are presented as the mean ± SD.

The capacitive performance of the FF/PPy-based SC in vitro was carried out in 1× PBS solution. The CV performance of FF/PPy was studied within the operating time from 0 to 21 days (Figure 4a). The capacitance of FF/PPy remains unchanged for 7 days, and the current density decreases to 30% of the initial value by day 14. At 21 days, the capacitor almost completely fails. The GCD curves were recorded under the current densities of 0.1 mA·cm^−2^ at the same time (Figure 4b). It shows the similar variation with CV curves. The mass specific capacitance of FF/PPy-based SCs during degradation are shown in Figure 4c. With the devices degraded in vitro, the mass specific capacitance is reduced. As shown in Figure S3d, the areal specific capacitance of SC changes from 26.8 mF·cm^−2^ to 3.25 mF·cm^−2^ for 21 days, which further indicates that the SC has a broad application prospect in the field of transient electronics. 

### 2.4. Biodegradability of SCs

As shown in Figure 4d and Figure S5, the SC is immersed in 1× PBS buffer solution for 90 days, and the biodegradation performance of SC recorded in real time. The capacitance performance of the device is maintained within 7 days, and the Mg electrode is corroded after 14 days, when cracks appear in the device. The structural integrity of the SC is maintained within 21 days, but the Mg electrode is completely degraded. At 35 days, the PLA–C substrate is decomposed into fragments due to hydrolysis reaction. With the increase in soaking time, the hydrolysis and bulk degradation becomes faster, the PLA–C membrane becomes powder, and the PVA/PBS hydrogel and FF/PPy active materials completely disappear (50 days). After 75 days, the device is almost completely degraded, indicating that the SC has good in vitro biodegradability under a normal physiological environment. At the same time, the mass loss during the degradation process was tested to study the degradation behavior of the material (Figure 4e). Initially, the mass loss can be attributed to the rapid dissolution of PVA/PBS hydrogel. Then, with the decomposition of PLA–C, Mg, and FF/PPy, and the repetitive replacement of the PBS solvent, the mass is further reduced. Finally, the SC is completely degraded after 90 days.

### 2.5. Biocompatibility of SCs

Biocompatibility is an important performance index of implantable devices, which is closely related to the component materials. In this study, L929 cells were used to evaluate the biocompatibility of FF/PPy films and SCs at the cellular level. L929 cells were cultured with DMEM medium that FF/PPy films and SCs were soaked in, As shown in Figure 4f and Figure S6, the attachment, proliferation, and morphological changes of cultured L929 cells at different times are observed by immunofluorescence staining. After 1 day, the cells in each group show good adhesion and single cell morphology. Subsequently, the cell density increases significantly with the extension of incubation time (2 days). By 3 days, some clusters of cells have formed and even grown into layers of confluent cells. The relative survival rate of L929 cells was determined by CCK-8 method (Figure 4g). The results are consistent with immunofluorescence staining. L929 cells have a high survival rate in 1–3 days (>95%), further demonstrating the biocompatibility of the device.

## 3. Discussion

In preliminary work, polypeptide materials have become a potential option for the next generation of flexible, light-weight, and pollution-free energy storage devices due to their good mechanical and electrical properties. However, the specific capacitance of the existing FF-based SCs should be improved, and the biosafety and environmental friendliness of the devices needed to be further verified. The FF/PPy-based SCs in the study demonstrate several unique advantages. Using PLA–C as the base, Mg as the electrode, FF/PPy as the active material, and PVA/PBS hydrogel as the electrolyte, the all-solid-state capacitor was assembled. The introduction of PPy not only stabilizes FF but also effectively improves the power storage capacity of FF, which greatly broadens the application scenario of the device. Moreover, all the constituent materials are proven to have good biosafety, light mass, and controllable size, which further promotes the batch applications of energy storage devices. In addition, all materials are degradable and can disappear into the environment in a short time, or be gradually degraded and absorbed in vivo once they have completed their tasks. These advantages make the eco-friendly SC an outstanding candidate for transient electronics. 

In summary, this work has made a detailed study on the electrochemical performance of FF, FF/PPy, and FF–Py. Among them, FF/PPy with 150 cycles of PPy deposition shows the best electrochemical performance, which can be attributed to the intertwined structure of the FF network and enhancement of conductivity of FF by the PPy deposition. A high capacitance retention of about 94.8% is achieved for FF/PPy-based SCs after 5000 cycles. The SCs can work stably for 7 days in a liquid environment and disappear completely within 90 days. This work promotes the development of biocompatible SCs with long-term stability and great environmental friendliness.

## 4. Materials and Methods

Synthesis of FF/PPy. PLA-–C films were cut into squares with the size of 2 cm × 2 cm and washed with ethanol in ultrasonic cleaner three times. After being dried in argon, they were sputtered with magnesium (Mg) on one side by magnetron sputtering devices (Denton Vacuum, Discovery 635) at 80 W for 30 min. Diphenylalanine (FF, 80 mg) was added into 10 mL 75% alcohol with hexafluoro–isopropanol (HFIP). After being ultrasonicated for 2 h, the dispersion of FF was obtained, and it was uniformly smeared on Mg/PLA–C plate with a volume of 1 mL. Then, pyrrole (Py, 0.01 mol) and potassium chloride (KCl, 0.1 mol) were added in deionized water (100 mL) [45]. After 1 h of ultrasonic treatment and 3 h of continuous stirring, the Py/KCl solution with a uniform appearance was obtained and applied as the electrolyte. The electropolymerization of Py was performed in a three-electrode electrochemical workstation (CHI6601 instruments, Shanghai, China) [46]. The as-prepared Mg/PLA–C plate with a size of 2 cm × 2 cm × 0.1 cm was used as the working electrode. A Pt sheet and Ag/AgCl were applied as the counter electrode and reference electrode, respectively. The electrodeposition cycles were 50, 100, 150, and 200 with a voltage window from 0 V to 0.8 V. After the electrodeposition, the cross-linked FF/PPy film were rinsed with distilled water and then dried in the air to measure the qualities.

Synthesis of FF–Py copolymer film. PLA–C films were cut into squares with a size of 2 cm × 2 cm and washed with ethanol in ultrasonic cleaner three times. After being dried in argon, the films were sputtered with Mg on one side by magnetron sputtering devices (Denton Vacuum, Discovery 635) at 80 W for 30 min. FF (80 mg) and Py solution were added into 75% alcohol. The mass ratios of FF and Py were 1:1, 1:2, and 2:1. The final volume of mixed solution was 10 mL. After being stirred uniformly for 1 h, the solution of the FF–Py copolymer was obtained. Then, it was evenly coated in Mg/PLA–C substrate with a volume of 1 mL and the film of FF–Py copolymer was fabricated after being dried in air.

Characterization of materials. The sample morphology and structure were characterized by scanning electron microscope (SEM, SU8020). Fourier transform infrared spectroscopy (FTIR spectra) were obtained with a FT-IR system (Bruker/VERTEX80v). Raman spectra were measured utilizing confocal microRaman spectrometer (LABRAM HR EVOLUTION). X-ray diffraction (XRD) study was performed using PANalytical XPert3 Powder with CuKα radiation (λ = 1.5406 Å). XRD patterns were recorded ranging from 5° to 35°. The voltage and current were 40 kV and 40 mA, respectively. 

Preparation of PVA/PBS hydrogel. The 10X PBS was purchased from Gibco^®^ by life technologies for cell culture and was diluted with deionized water to achieve a proper concentration (1×, 0.01 mol·L^−1^). A total of 2 g of PVA powder was dissolved in the as-prepared PBS (1×, 20 mL) by sonication at 80 °C until it formed a homogeneous sticky solution. Then, after the solution was cooled down to room temperature, the clear and transparent hydrogel was prepared.

Assembly of all-solid-state symmetric supercapacitors. Two pieces of Mg/PLA-C substrate with FF/PPy film deposited on them and a PVA hydrogel were assembled immediately into a sandwich structure. Several drops of PVA solution (0.2 mL) were added onto the dried PVA/PBS hydrogel film to make the hydrogel fit with the FF/PPy film. After being clamped with an alligator clip for about one hour, the extruded PVA hydrogel around the SC edges was completely dried out and the all-solid-state symmetric supercapacitor was assembled. The SCs based on FF–Py copolymer films and FF films had the same preparation processes and structure.

Electrochemical measurements. The electrochemical properties of the SCs were measured by a CHI6601 electrochemical workstation, including cyclic voltammetry (CV) curve, galvanostatic charge/discharge (GCD), and electrochemical impedance spectroscopy (EIS). The CV curves were measured under different scan rates from 10 to 100 mV·s^−1^ between 0 V and 0.8 V. EIS measurements were carried out in the frequency range from 100 kHz to 0.01 Hz at open circuit potential with an AC perturbation of 5 mV. Electrochemical measurements were carried out in 1.0 M KCl aqueous solution at room temperature. The specific capacitance was recalculated to mF·cm^−2^ according to the following Formula (1) [47] and (2) [48,49,50].

(1) $$C_{a}=\frac{\int IdV}{v\cdot \Delta V\cdot A}\;$$

(2) $$C_{s}=\frac{2I\times \Delta t}{m\times \Delta V}\;$$

where *C_a_* (mF·cm^−2^) is the areal specific capacitance, *C*_s_ (F·g^−1^) is the mass specific capacitance, *I* (A) is the response current, *v* (V·s^−1^) is the voltage scan rate, *m* (g) is the active material mass on one electrode, Δ*V* (V) is the voltage window, and *A* (cm^2^) is the effective area of active material (FF/PPy or FF–Py copolymer) layer.

Dissolution tests for supercapacitors. To investigate the dissolution process and dynamics of FF/PPy-based SCs, we performed a series of dissolution tests in 10 mL of 1× PBS buffer at room temperature for 90 days. In the dissolution tests, FF/PPy-based SCs with an original size of 1 cm × 1 cm were regularly taken out from PBS solution, rinsed in DI water, and then dried to measure the weight. After that, a fresh PBS solution was used to ensure the same chemical environment every day. 

Cellular viability test. The FF/PPy film and FF/PPy-based SCs were soaked in DMEM culture medium for 3 days and filtered to obtain supernatant culture medium. Then, L929 cells (5 × 10^3^ cells per well, 100 μL per well) were cultured with the above medium and incubated at a humidity atmosphere containing 5% CO_2_ at 37 °C for 1, 2, and 3 days. Cell viability and proliferation of L929 cells were evaluated by CCK-8 assay. After co-culturing for 1, 2, and 3 days, 10 μL of CCK-8 solution was added to each well and then incubated for 4 h in a humidified atmosphere with 5% CO_2_ at 37 °C. The optical density value at a wavelength of 450 nm was measured with a multimode microplate reader (ThermoFisher Multiskan FC, Milton Freewater, OR, USA). Three parallel control cells were applied to each group.

Cell morphology and immunofluorescence staining. After the supernatants were removed at the time of 1, 2, and 3 days, the L929 cells were washed with 1× PBS buffer three times at room temperature and then stained with calcein-AM and propidium iodide (PI) for 30 min and 5 min, respectively. The immunofluorescence images were taken using a Leica confocal fluorescence microscope (LECIA TCS SP8) in 490 ± 10 nm and 545 nm emission filter.

## Data Availability

Data sharing not applicable as no datasets have been generated or analyzed in this study.

## Supplementary Materials

The following supporting information can be downloaded at: https://www.mdpi.com/article/10.3390/ijms24032497/s1.
