# Supplementary material for: Polypyrrole-Stabilized Polypeptide for Eco-Friendly Supercapacitors
Source: Int J Mol Sci. 2023 Jan 27;24(3):2497. doi: 10.3390/ijms24032497 (PMC9916972; doi:10.3390/ijms24032497)
Supplement: Supplementary file 1 [file ijms-24-02497-s001.zip › ijms-2164291-supplementary.pdf]

Supplementary Materials: Polypyrrole-Stabilized Polypeptide for Eco-Friendly Supercapacitors

Zhe Li, Kuan Hu, Zhou Li, Cong Li and Yulin Deng

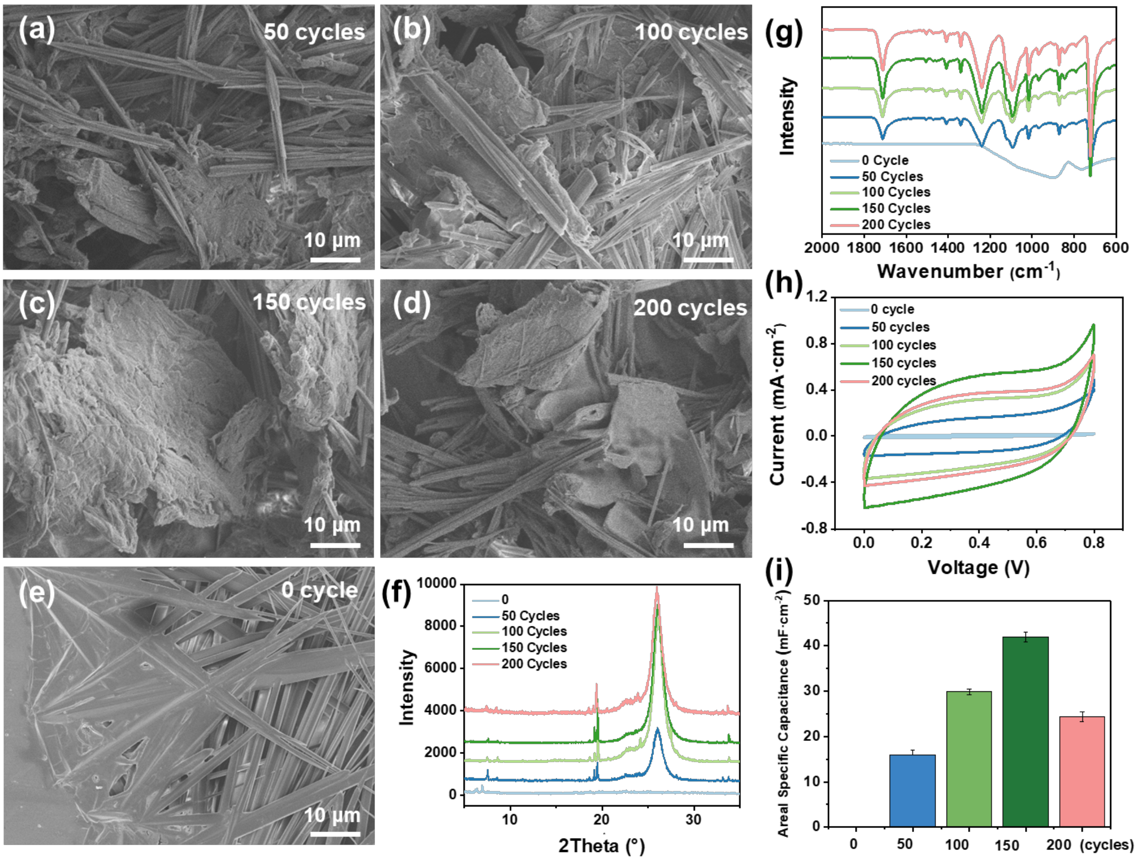

Figure S1. Characterization and electrochemical property of FF/PPy films with different deposition cycles. (a-e) Micro morphologies of FF/PPy with different electrodeposition cycles. (a) 50 cycles; (b) 100 cycles; (c) 150 cycles; (d) 200 cycles. (e) SEM image of FF (0 cycle). (f) XRD patterns. (g) Fourier infrared spectrum (FTIR). (h) CV curves at 100 mV·s-1; (i) Areal specific capacitances calculated from the CV curves.

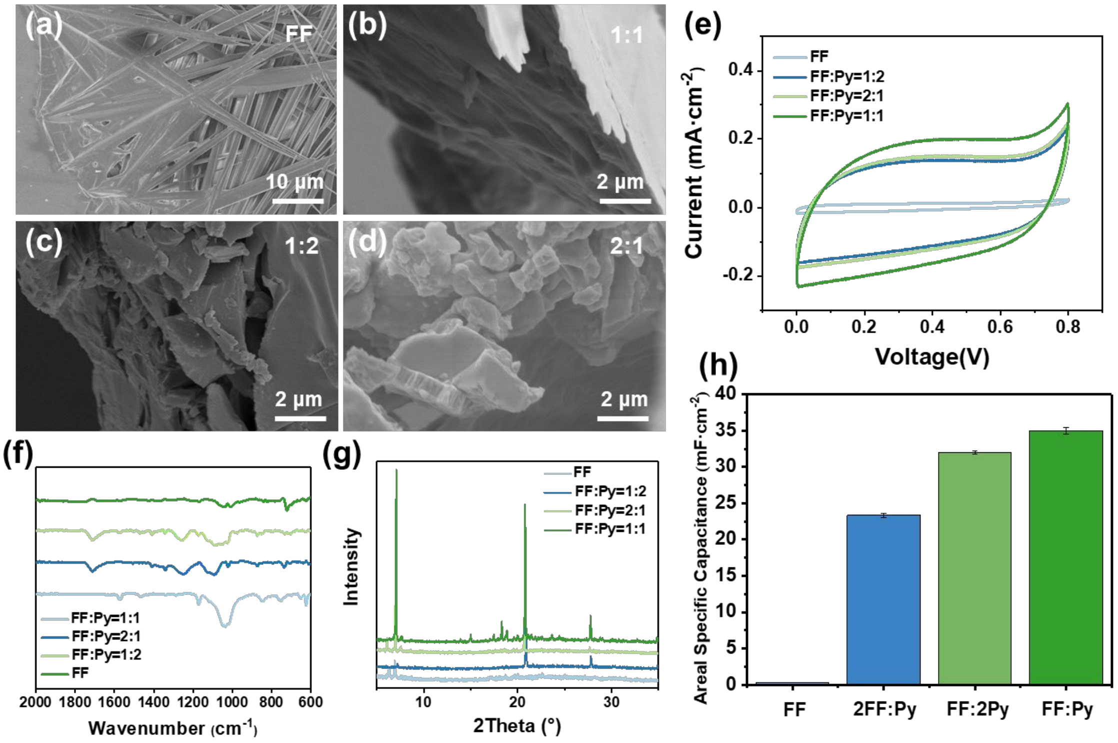

Figure S2. Characterization and electrochemical property of FF–Py copolymers with different ratio of FF and Py. (a-d). SEM images of FF–Py copolymers with three different ratios of FF and Py. (a) Pure FF; (b) FF:Py = 1:1; (c) FF:Py = 1:2 and (d) FF:Py = 2:1. (e). FTIR spectrum. (f). XRD pattern. (g). CV curves. (h). Areal specific capacitance.

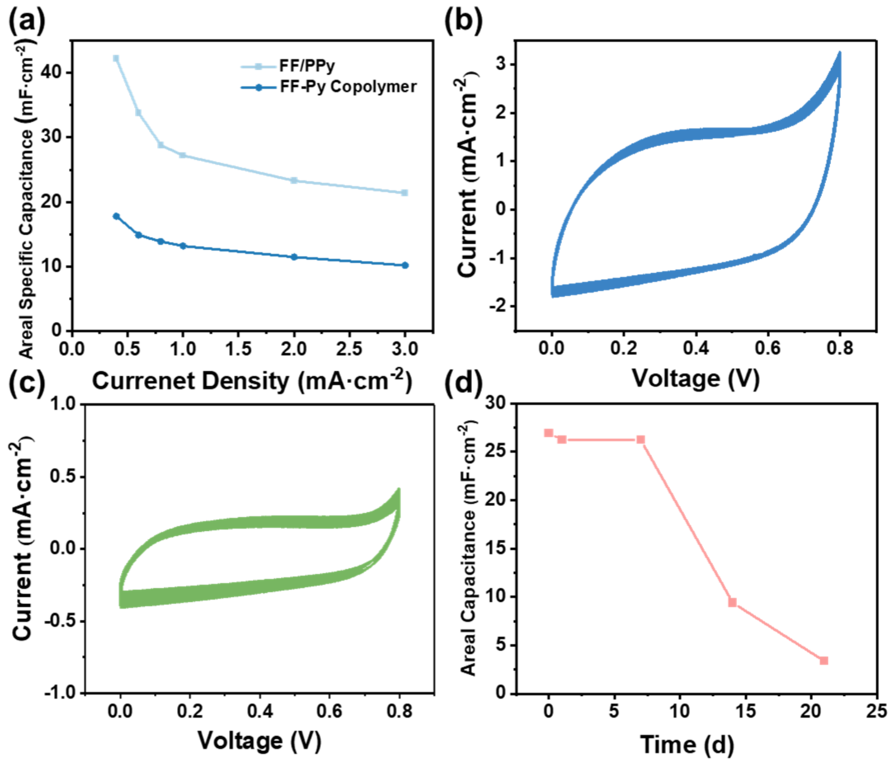

Figure S3. (a) Areal specific capacitances of SCs based on FF/PPy and FF–Py copolymer films with different scan rates; cycle stability test of SCs based on FF/PPy (b) and FF–Py copolymer (c) films with the current density of 0. 2 mA·cm^{-2} at 0.8 V; (d) the variation of areal specific capacitances during the degradation.

Table S1. A comparison with recently developed peptide-based nanomaterials and hybrids electrode materials and electrochemical performances.

Electrode Materials	Electrolyte	Specific Capacitance	Current Density/Scan Rate	Cycle Number & Capacitance Retention	Degradable	Ref.
Glassy carbon with peptides BDCP-1-R	0.05M KH_2PO_4 and 0.5MKCl	2.35 $mF{\cdot}cm^{-2}$	50 $mA{\cdot}cm^{-2}$	80% after 5000 cycles at a scan rate of 0.4 $mV{\cdot}s^{-1}$	No	[21]
Fmoc-EF-NH_2 peptide@TiO_2	PVA/H_3PO_4 gel	8.6 $mF{\cdot}cm^{-2}$	10 $mV{\cdot}s^{-1}$	> 95% after 5000 cycles	No	[29]
Benzo selenadiazole-5-carbonyl-protected dipeptide cross-linked with cobalt hydroxide (BSeYY/$Co(OH)_2$) on carbon fiber paper	1 M KOH and 1 M LiOH	974.78 $F{\cdot}g^{-1}$	1 $A{\cdot}g^{-1}$	78.62% after 3000 cycles	No	[33]
Ni foam with uniform branched MnO_2/peptide hybrid nanowires	1 M Na_2SO_4	421 $F{\cdot}g^{-1}$	1 $A{\cdot}g^{-1}$	93% after 2500 cycles	No	[34]
Peptide coated Toray carbon paper	0.1 M K_2SO_4 at pH 7.4 and 0.1 M KCl	2210 $\mu F{\cdot}\ cm^{-2}$	50 $mV{\cdot}s^{-1}$	500 cycles	No	[35]
Diphenylamine (FF) peptide nanotubes-coated carbon	1 M H_2SO_4	800 $mF{\cdot}cm^{-2}$	100 $mV{\cdot}s^{-1}$	-	No	[36]
Aromatic dipeptide nanotubes-modified carbon	0.05 M KH_2PO_4 and 0.5 M KCl	120 $\mu F{\cdot}cm^{-2}$	100 $mV{\cdot}s^{-1}$	-	No	[37]
FF peptide-modified carbon	0.05 M KH_2PO_4 and 0.5 M KCl	240 $\mu F{\cdot}cm^{-2}$	-	-	No	[38]
Aromatic diphenylalanine (FF)-based nanohybrid BSeFF/$Ni(OH)_2$ –	1 M KOH	1250 $F{\cdot}g^{-1}$	2 $A{\cdot}g^{-1}$	72% after 1000 cycles	No	[39]

deposited nickel foam						
FTO electrode coated with unidirectionally aligned FF NTs/MTs	0.05 M KH_2PO_4 and 0.5 M KCl	1000μF·cm^{-2}	50 mV·s^{-1}	90% after 5000 cycles	No	[40]
Activated carbon	KOH-saturated mCel-membrane	153.34 mF· cm^{-2}	10 mV·s^{-1}	84.7% after 10000 cycles	No	[41]
Peptide-Co_9S_8 nanobricks	KOH/PVA hydrogel	1.3 F·cm^{-2}	0.7 mA·cm^{-2}	96% after 5000 cycling	No	[42]
Water-soluble metal (W, Fe, Mo)	Agarose gel	1.6 mF· cm^{-2}	0.15 mA· cm^{-2}	-	Yes	[11]
Activated charcoal	Gatorade	78.8 F·g^{-1}	1 A·g^{-1}	92.3% after 1000 cycles	Yes	[43]
Fe-ZnO on PLA film	PVA/PBS hydrogel	0.36 mF· cm^{-2}	10 mV·s^{-1}	70% after 3000 cycles	Yes	[44]
FF/PPy coated Mg/PLA-C plate	PVA/PBS hydrogel	42.6 mF· cm^{-2}	0.4 mA·cm^{-2}	94.8 % after 5000 cycles	Yes	This work

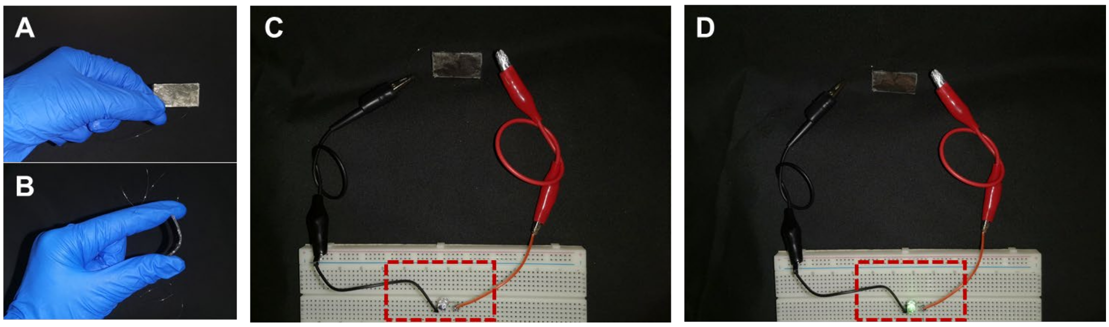

Figure S4. Pictures of freshly fabricated FF/PPy-based SCs and practical demonstration using SCs to light up the green LED pattern. (A) The picture of a SC. (B) The SC is flexible. LED (C) before and (D) after power supply with FF/PPy-based SCs.

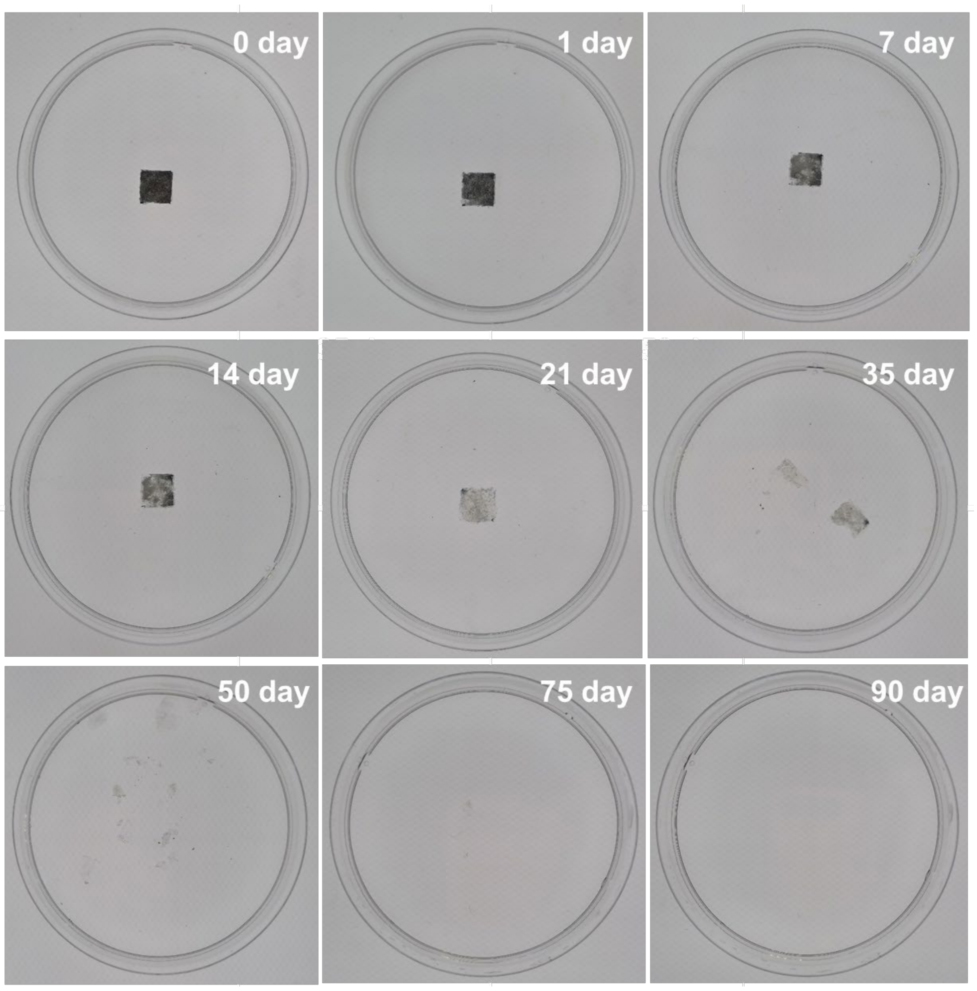

Figure S5. In vitro degradation of SCs in 1X PBS buffer at room temperature for 90 days.

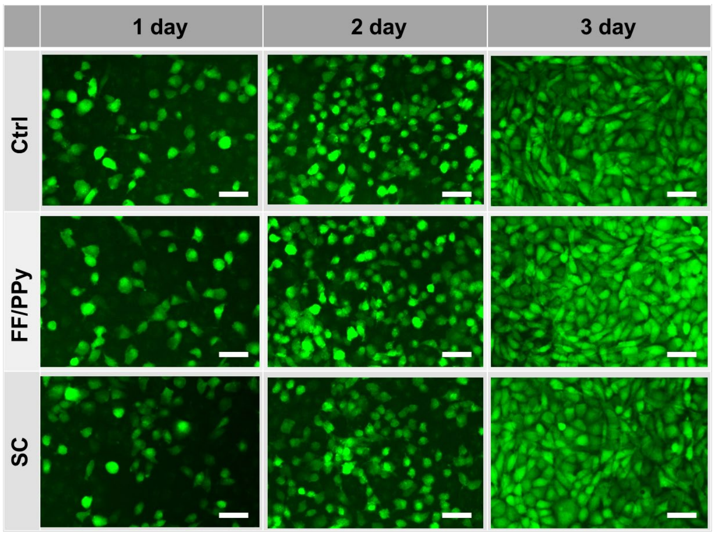

Figure S6. Biocompatibility of FF/PPy film and SC. Attachment, proliferation, and morphology of the L929 cells at different times. Scale bars: 20 μm.
